# Sorting of complex sphingolipids within the cellular endomembrane systems

**DOI:** 10.3389/fcell.2024.1490870

**Published:** 2025-02-26

**Authors:** Victor O. Svistunov, Kigumbi J. Ehrmann, Wayne I. Lencer, S. S. Schmieder

**Affiliations:** ^1^ Division of Gastroenterology, Hepatology and Nutrition, Boston Children’s Hospital, Boston, MA, United States; ^2^ Division of Gastroenterology, Hepatology and Nutrition, Boston Children’s Hospital, Harvard Medical School, Boston, MA, United States; ^3^ Department of Pediatrics, Harvard Digestive Diseases Center, Boston, MA, United States

**Keywords:** membrane nanodomains, endocytosis, membrane trafficking, membrane curvature, membrane rafts, complex sphingolipids

## Abstract

Cells contain a plethora of structurally diverse lipid species, which are unevenly distributed across the different cellular membrane compartments. Some of these lipid species require vesicular trafficking to reach their subcellular destinations. Here, we review recent advances made in the field that contribute to understanding lipid sorting during endomembrane trafficking.

## 1 Introduction

The plasma- and endo-membranes of eukaryotic cells are two-dimensionally diffusing fluids comprised of myriads of lipid species. They form a barrier, separating compartments into selective reaction spaces, and embed the trans- and membrane proteins of the cell. It is estimated that each cell consists of tens of thousands of lipid species (Gerl et al., 2012) with each cellular membrane compartment or organelle having its unique and distinct lipid composition and thus membrane identity (Harayama and Riezman, 2018; van Meer et al., 2008). The different membrane and organelle compartments, however, are interconnected through vesicular transport and thus in constant exchange. For the plasma membrane (PM), for instance, it is estimated that the equivalent of its total surface area is turned over every 15 min (Koval and Pagano, 1991; Mayor et al., 1993). Given this continuous vesicular endomembrane flux along the endocytic and secretory systems, concomitant lipid and protein sorting are thus key cellular processes necessary to enable eukaryotic cells to maintain membrane homeostasis among organelles (van Meer et al., 2008).

There is an increasing body of research demonstrating how individual or bulk lipids can be supplied to different organelles through either lipid transfer proteins specific for individual lipids or through membrane contact sites that connect different organelles. Both pathways bypass vesicular trafficking (interested readers might be referred to the following references: Anders and Mattjus, 2021; Khaddaj and Kukulski, 2023; Melia and Reinisch, 2022; Samaha et al., 2019; Wong et al., 2019).

However, these lipid transport conduits are not available for all lipid species. Due to their large hydrophilic headgroup, complex sphingolipids (cSLs), sphingomyelin and especially the glycosphingolipids (GSLs), are trapped in the outer membrane leaflet and cannot rely on lipid transfer proteins for sorting (Sokoya et al., 2022; van Meer et al., 2008; Young et al., 1992). Rather, complex SLs depend heavily on vesicular trafficking to reach their subcellular destinations. The mechanisms by which cells preferentially sort these lipids during trafficking to their respective compartments and organelles, and the underlying biophysical driving forces, remain open questions.

This is the topic of this review: to collect our current understanding about the mechanisms by which cells can sort their plethora of different SL species that rely on vesicular trafficking to maintain compartment and organelle homeostasis.

## 2 Lipid self-organization and membrane nanodomains

**FIGURE 1:**
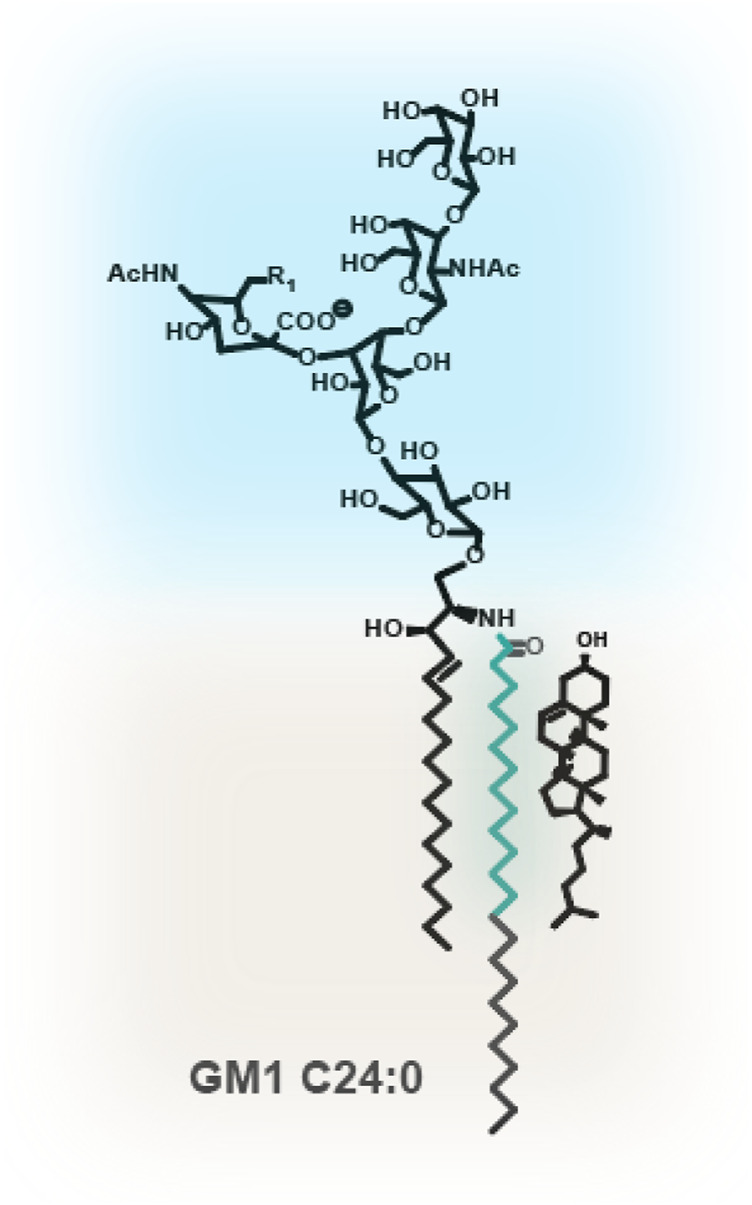
Glycosphingolipid GM1. GSLs such as GM1 contain a large and hydrophilic oligosaccharyl headgroup, protruding into the extracellular space. The ceramide is composed of a C18 or C20 sphingoid base, containing a trans double carbon bond at C4. The acyl chains can vary dramatically in length and degree of unsaturation. Depicted is a C24 fully saturated acyl chain. In turquoise is the C14* motif. 14 fully saturated hydrocarbons from the amide bond at the water-bilayer interface are required for assembly with cholesterol.

How membrane lipids interact with each other is critical for vesicular-based lipid sorting. Membrane lipids, including SLs, are structurally extremely diverse. This diversity stems from different headgroups, e.g., for complex sphingolipids, this can be a choline (sphingomyelin), or a diversity of different sugar headgroups for the glycosphingolipids. Phospholipids occur as phosphatidylcholine (PtdCho), -ethanolamine (PtdEtn) or -serine (PtdSer), but all lipids also differ in their acyl chain structures. The acyl chains of lipids can vary in their hydrocarbon chain length and in their degree of saturation. Most phospholipids are ‘hybrid’ lipids and contain one saturated acyl chain at the sn1 position and one cis mono- or polyunsaturated fatty acid at their sn2 position. Phospholipids with two saturated or two unsaturated acyl chains are relatively scarce. Sphingolipids on the other hand, are anchored in the membrane through a ceramide portion, characterized by a long chain sphingoid base connected to an acyl chain of varying length, between 14 and typically 24 hydrocarbons. The sphingoid base is usually 18 or 20 hydrocarbons in length and comprises a trans carbon double bond at C4. The acyl chain, however, is predominantly saturated or contains one cis carbon double bond (Figure 1; Hannun and Obeid, 2018; Merrill, 2011).

**FIGURE 2:**
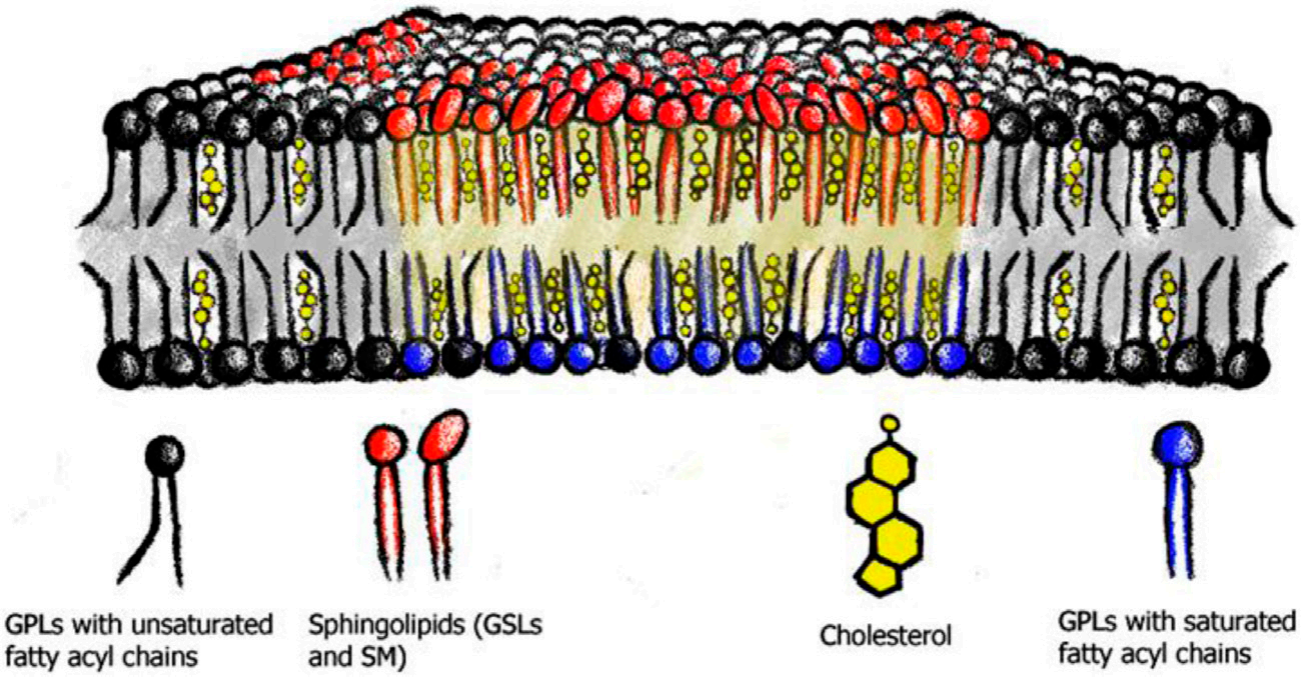
Membrane nanodomains or lipid rafts. Saturated phospholipids (blue), sphingolipids (red) and cholesterol (yellow) assemble into membrane nanodomains or lipid rafts. These phase separate from the phospholipids with predominantly unsaturated acyl chains (black).

There are two ways in which lipids can organize in the membrane, first by shaping the membrane physically or by creating lateral heterogeneity. The individual attributes of a lipid, the ratio between the acyl chain structures, and the size of a lipid’s headgroup give lipids an intrinsic shape or geometry (Pinot et al., 2014; Luzzati et al., 1966; Luzzati et al., 1968; Florence et al., 2004; Vanni et al., 2014; Maggio et al., 1978; Cebecauer et al., 2018; Frolov et al., 2011). Each cis carbon double bond induces a kink in the acyl chain tail, thus requiring more physical space than its saturated counterpart and therefore reducing the ability of the lipid to pack side-by-side (Chiantia et al., 2006; Dietrich et al., 2001; Hitchcock et al., 1974; McIntosh and Simon, 1986; Olbrich et al., 2000). Additionally, the two acyl chains can vary in hydrocarbon chain length, for some requiring interdigitation into the opposing leaflet. Apart from the acyl chain structure, the size of the head group affects the lipid’s overall shape (Rawicz et al., 2000). Phosphatidylcholine (PtdCho), phosphatidylserine (PtdSer) and the sphingolipid, sphingomyelin are cylindrical lipids, while lipids such as phosphatidylethanolamine (PtdEtn), phosphatic acid, and diacylglycerol (DAG) or cholesterol, have a smaller polar headgroup, and thus adopt a conical shape. GSLs, with their large oligosaccharyl headgroup adopt an inverse conical shape, with the headgroup requiring more space than the ceramide (Hitchcock et al., 1974). A lipid’s structural features (both the acyl chain structures and size of the headgroup) also critically influence its ability to interact with other lipids and pack side-by-side (see below for the concept of membrane nanodomains, rafts and the liquid-ordered phase). They give rise to small lateral inhomogeneities within the membrane space, where lipids are not uniformly distributed or ‘mixed’, but instead can be highly organized and form membrane domains with distinct lipid compositions (Figure 2; Heberle and Feigenson, 2011; Almeida, 2009).

One example of how lipids can self-associate is the concept of membrane nanodomains and lipid rafts (Simons and Ikonen, 1997). These nanodomains or rafts are membrane areas enriched in cholesterol, saturated lipids, and especially sphingo- and glycosphingolipids, and are thought to organize the plasma membrane into heterogeneous sub-domains, to compartmentalize cellular functions (Brown and London, 1998a; Pike, 2006), e.g., immune signaling (Dinic et al., 2015; Field et al., 1995; Gupta and DeFranco, 2007), endocytosis (Kim et al., 2017), host-viral/toxin interaction processes (Chinnapen et al., 2007; Dick et al., 2012; Johannes, 2017; Wang et al., 2008), and protein clustering (Arumugam et al., 2021). Importantly, another physiological role for nanodomains is believed to be the sorting and trafficking platform for membrane components between subcellular organelles (Diaz-Rohrer et al., 2014; Schuck and Simons, 2004; Smart et al., 1996).

The lipid raft hypothesis was originally conceived to explain differences in membrane sorting between the apical and basolateral membranes of polarized epithelial cells (Simons and Ikonen, 1997), but the overall concept was already suggested earlier by Stier and Sackmann (1973). It assumes that rafts or nanodomains have different physical properties by creating a highly packed and ordered lipid environment. This leads to an altered membrane miscibility, with increased membrane thickness and rigidity and a reduced diffusiveness of its components (Lingwood and Simons, 2010; Simons and Sampaio, 2011; Sezgin et al., 2017; Simons and Toomre, 2000; Cebecauer et al., 2018). The nanodomain or raft concept is supported by a wealth of *in vitro* studies on model membrane systems, where SLs, phospholipids with saturated acyl chains, and cholesterol phase-segregate into liquid-ordered (Lo) regions, with tight lipid-lipid packing, due to their preferred interactions. Similar to the liquid-disordered (Ld) phase, the Lo phase is still fluid, allowing molecular motion of the individual components, albeit at reduced diffusiveness. The Ld phase is characterized by weak lipid-lipid packing, higher permeability, and low membrane rigidity. The distinctiveness between these two phases allows them to coexist over a large compositional spectrum (Bacia et al., 2005; Brown and London, 1998b; Feigenson, 2009; Hjort Ipsen et al., 1987; Sezgin et al., 2012; Simons and Vaz, 2004; Veatch and Keller, 2005; Wesołowska et al., 2009).

In addition, the concept is supported by many atomistic simulations characterizing cholesterol interactions in membrane bilayers, with favorable packing between cholesterol and saturated lipid acyl chains (Martinez-Seara et al., 2010; Róg et al., 2007).

While we have a good understanding of the physicochemical principles that drive phase separation and raft formation in artificial membrane systems, their existence, relevance, and locations in live cells remain controversial to this day. Evidence for macroscopic phase separated Lo domains in live cells comes predominantly from work on the vacuole of the budding yeast *Saccharomyces cerevisiae*. Here, the vacuolar membrane and membrane associated proteins start to phase separate when the yeast is entering the stationary growth phase. These vacuolar Lo domains show similar characteristics than what is observed in GUVs (Leveille et al., 2022; Moeller and Thomson, 1979; Moeller et al., 1981; Toulmay and Prinz, 2013). Interestingly, in a recent study, Kim et al. could demonstrate that this vacuolar phase separation is driven by a change in lipid trafficking and thus resulting redistribution of cellular complex SL into the vacuole (Kim and Budin, 2024).

Instead of large macroscopic phase separated domains, mammalian live cell plasma and endomembranes are thought to contain small membrane nanodomains, which are highly dynamic and typically less than 20 nm in size (Kenworthy and Edidin, 1998; Lagerholm et al., 2005; Lingwood and Simons, 2010; Nichols, 2003; Sharma et al., 2004; Varma and Mayor, 1998; Veatch and Keller, 2003; Hancock, 2006; Levental et al., 2020). Direct evidence for their existence comes from studies investigating the differential behavior and dynamics of, e.g., fluorescently labeled lipids or GPI-anchored proteins (Eggeling et al., 2009; Kinoshita et al., 2017; Komura et al., 2016; Mueller et al., 2011; Saha et al., 2016; Stone et al., 2017; Honigmann et al., 2014). This includes our own work, where, using a GSL library with varied ceramide structures in live cells, we found evidence that incorporation of GSLs into membrane nanodomains requires a specific number of saturated carbon atoms. We termed this motif within the acyl chain the “C14* motif” (Figure 1). This stretch of 14+ saturated hydrocarbons from the amide bond at the water-bilayer interface most likely represents the minimal motif within an acyl chain to associate and accommodate cholesterol packing (Arumugam et al., 2021; Schmieder et al., 2022). While many studies, including our own, have demonstrated a necessity for cholesterol in nanodomain formation, the Kraft group, interestingly, using a technique called NanoSIMS, could not detect such cholesterol-sphingolipid domains in the PM; instead, they observed local sphingolipid-exclusive enrichments (Yeager et al., 2016).

**FIGURE 3:**
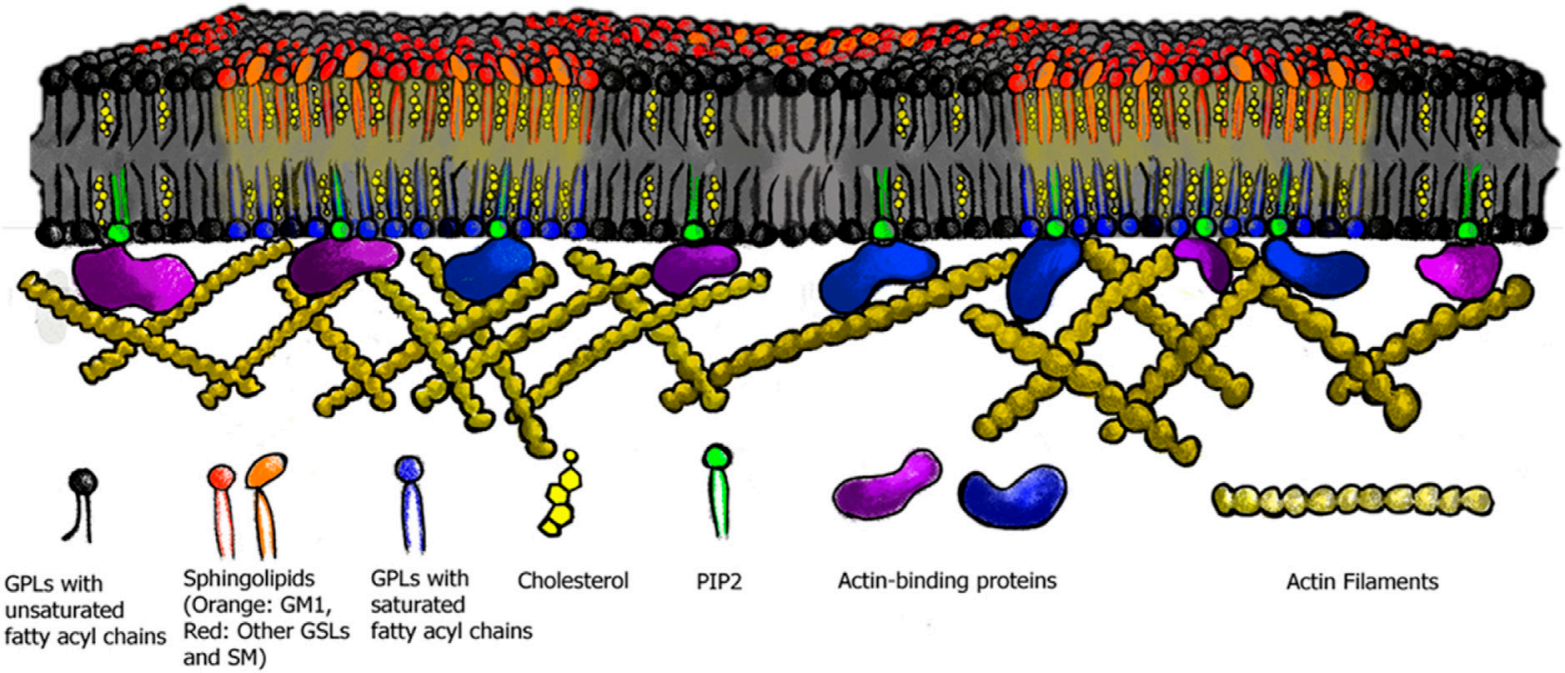
Membrane nanodomains in live cells. The actin cytoskeleton, as well as membrane associated proteins are thought to stabilize and affect nanodomain size in live cell membranes. Membrane proteins which bind to PtdSer or PtdIns crosslink the membrane to the cortical actin cytoskeleton.

Additionally, a restriction for macroscopic phase separation in the PM of live cells, is presumably the dynamic cortical actin cytoskeleton, which likely affects the location, size, and timing of nanodomain domain formation (Badizadegan et al., 2000; Kraft, 2016; Kusumi et al., 2005a; Liu and Fletcher, 2006). Specifically, nanodomains in the outer membrane leaflet are coupled to the preexisting actin-myosin networks inside the cell, which are mediated by the inner leaflet lipid PS (Raghupathy et al., 2015). Molecular simulations, e.g., demonstrated that filamentous supports, modeling the cortical actin cytoskeleton, coupled to lipids, have the potential to segregate membranes into corrals and stabilize domain formation, even at relatively low connectivity to the membrane, supporting the picket-fence model of membrane organization through the actin cytoskeleton (Levental et al., 2020; Tsai et al., 2024; Kusumi et al., 2005b; Kusumi et al., 2004 Kusumi et al., 1999) (Figure 3). Furthermore, extracellular binding to and cross-linking of nanodomain components by, e.g., endogenous lectins or exogenous toxins, which bind and crosslink the extracellular headgroup of especially GSLs, can result in stabilization and/or coalescence of membrane nanodomains and thereby affect their lifetime and function (Roemer et al., 2007; Szklarczyk et al., 2013; Hammond et al., 2005; Arumugam et al., 2021; Koyama-Honda et al., 2020; Day et al., 2015; Garner and Baum, 2008; Johannes et al., 2018; Raghunathan et al., 2016).

## 3 Lipid landscape of a cell

The lipid landscape of a cell, meaning the distinct membrane compositions of cellular organelles, has been described by the concept of evolutionarily conserved ‘lipid territories,” delineating the organelle and vesicular intermediates as two ends of distinct lipidome spectrums, with a “PM territory” on the one side and an “ER territory” on the other (Bigay and Antonny, 2012; Kim and Burd, 2023). The “plasma membrane (PM) territory” comprises the plasma membrane itself, the trans Golgi network (TGN), as well as the secretory and endolysosomal networks, while the endoplasmic reticulum (ER), the cis, and medial cisternae of the Golgi apparatus belong to the “ER territory.” The distinction between these two membrane territories is based on differences in a) lipid compositions, especially SL and cholesterol, leading to differences in b) membrane order, while also differing in c) the net charge of the leaflets and d) the degree of lipid species asymmetry between the bilayer leaflets (Holthuis and Menon, 2014; Kim and Burd, 2023). The membranes of the “ER territory” are characterized by a low membrane order due to a relative absence of nanodomain forming lipids, specifically in SL but also cholesterol (van Meer et al., 2008). The “PM territory,” on the other hand, arises from the synthesis of nanodomain-forming SLs and a consequent sequestration and enrichment of cholesterol within the late Golgi compartments due to their preferential interactions (Orci et al., 1981; Hanada et al., 2003; Sharpe et al., 2011; Slotte, 2013). SLs and cholesterol enrich gradually in the outer membrane leaflets of the secretory pathway leading to the plasma membrane. Their assembly into nanodomains leads to a high degree of packing order characterizing this membrane territory. Sequestration of cholesterol and SL in the outer membrane leaflet and the presence of PtdSer and phosphatidyl inositol (PtdIns) species on the cytoplasmic leaflet give rise to a highly asymmetric membrane. The increased order and associated increase in membrane thickness allow for the required barrier function in the PM. The gradual increase in membrane thickness through the synthesis of SLs within the Golgi and TGN and the following increase in cholesterol have been hypothesized to be a means to sort PM proteins in the Golgi for PM delivery by hydrophobic mismatch (Bigay and Antonny, 2012; Kim and Burd, 2023) of the transmembrane domain with membrane thickness. Indeed, the transmembrane domains of PM-resident transmembrane proteins contain slightly longer TMs and generally sort into Lo domains (Lorent et al., 2017; Munro, 1995; Quiroga et al., 2013).

The differences in SL and GSL composition between the two membrane territories are believed to rely on the differential SL trafficking and sorting between them. Importantly, the physico-chemical features of these territories seem to be conserved throughout eukaryotic evolution (Bigay and Antonny, 2012; Kim and Burd, 2023).

The importance of the SL gradient in organelle identity and its maintenance was recently illustrated by a study by Sokoya et al. (2022). Here, the sphingomyelin gradient in the secretory pathway was disrupted by the mislocalization of sphingomyelin synthase to the ER due to a pathogenic mutation. The subsequent synthesis of sphingomyelin in the ER and lack thereof in the TGN and PM resulted in manifold changes in the overall amounts of many different lipid species, with altered overall membrane lipid packing within the secretory pathway, and aberrant cholesterol accumulation in cytoplasmic vesicles, leading to osteoporosis and skeletal dysplasia in the patients (Pekkinen et al., 2019; Sokoya et al., 2022).

## 4 Lipid sorting principles

**FIGURE 4:**
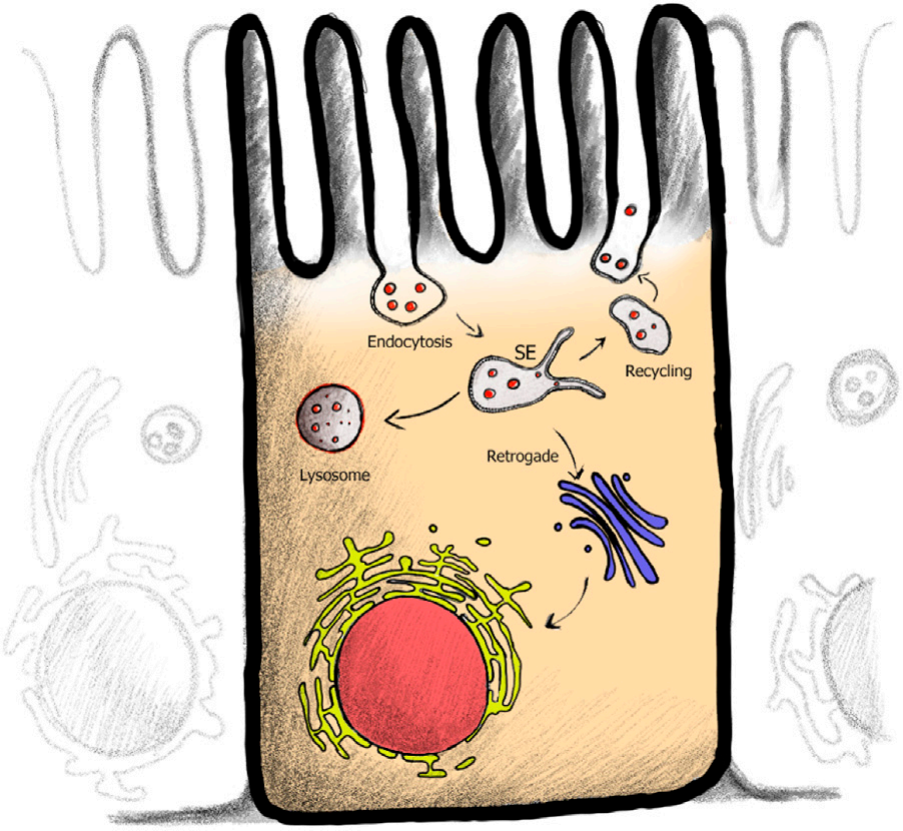
Endocytic membrane trafficking pathways within cell. After plasma membrane (PM) cargo is endocytosed, cargo is sorted within the sorting endosome (SE). Pathway specific tubules are pulled from the SE, serving the recycling (back to the PM), retrograde (PM to Golgi to ER) or in polarized epithelial cells the transcytotic (linking apical and basolateral membranes) pathways. Cargo destined for degradation remains in the vesicular part of the endosome.

In the previous section, we provided an overview of the SL and GSL distributions across the two different cellular lipid territories. Without lipid sorting, however, vesicular trafficking, interconnecting the different territories, would quickly erode this gradient. How can cells maintain SL and GSLs compositions across their organelles without re-distribution by lipid transfer proteins or through membrane contact sites? There is compelling evidence that lipids are sorted differentially into transport carriers in live cells for both the secretory and endocytic pathways (Figure 4).

While SL synthesis starts with ceramide production in the ER, ceramide itself is trafficked to the Golgi by both vesicular trafficking and ceramide-specific transport proteins (Funato and Riezman, 2001). Only the addition of headgroup in the Golgi lumens, especially the large and hydrophilic oligosaccharide of GSLs destines SL and GSLs to vesicular trafficking for sorting. Recent studies in both polarized epithelial cells and nonpolarized cells show sphingomyelin sorting and enrichment into specific TGN-derived vesicles, thus supporting the evidence of Golgi-to-PM lipid sorting for SL and cholesterol (Deng et al., 2016; Klemm et al., 2009; Meer and Sprong, 2004; Wakana et al., 2020).

Early studies showed that, within the endosomal system, saturated and thus nanodomain-forming lipids were depleted from endosomal recycling tubules compared to unsaturated lipids (Gruenberg, 2003; Maxfield and McGraw, 2004; Mukherjee et al., 1999; Mukherjee and Maxfield, 2000). And within the retrograde pathways (trafficking from the PM to the Golgi and back to the ER), COPI-coated vesicles were found to be depleted of SLs (Brügger et al., 2000; Manneville et al., 2008). These results are in line with our own observations utilizing a GSL library of different ceramide structures. GSL species lacking a C14* motif and thus unable to form membrane nanodomains (Figure 1) were found in endosomal sorting tubules of the recycling, the retrograde, as well as in polarized epithelial cells transcytotic pathways (Schmieder et al., 2022; te Welscher et al., 2014; Chinnapen et al., 2012; Chinnapen et al., 2012). However, GSL species containing a C14* motif, which enables incorporation of the GSL into membrane nanodomains, were instead significantly depleted from these pathways and were sorted instead into the degradative pathway. Additionally, we could identify distinct lipid domains within enlarged endocytic carriers, where C14* motif containing GM1 species were segregated from transferrin receptor positive and C14* motif-lacking GM1 species (Schmieder et al., 2022).

### 4.1 Curvature based lipid sorting

Apart from lipid composition, membrane curvature also changes throughout the endomembrane compartments, suggesting a role for curvature as a means of sorting lipids (Black et al., 2013). Membrane vesicles, which facilitate inter-organelle traffic, are produced by budding and fission of the membrane from a donor organelle. This induction of highly curved membranes is thought to facilitate sorting of lipids.

Over the years, there has been a wealth of *in vitro* evidence to support how individual lipid species can be preferentially sorted through a curvature-based sorting mechanism. Initially, lipid shape was thought to be a prime candidate for how lipids might be sorted across membrane curvature. This was based on the idea that lipids might distribute spontaneously to differentially curved membrane regions according to the intrinsic geometrical shape of the lipid (see concept above, C. Black et al., 2014; Cheney et al., 2017; Hatzakis et al., 2009; Lodish, 2008). The molecular basis of this argument was that specific lipid species are not cylindrical-shaped but are conical and/or inverse-conical, and therefore would preferentially sort into membrane areas with curvature that accommodates and complements their shape. For instance, lipids with an inverse conical shape, comprising lipids with a large headgroup to acyl chain ratio, e.g., lysoPC with a single tail and a large headgroup, PtdIns or the GSLs, favor membrane regions of positive curvature bending the monolayer away from their large headgroups (reviewed in: Chernomordik and Kozlov, 2003; Di Paolo and De Camilli, 2006; Zimmerberg and Kozlov, 2006). On the other hand, lipids with both acyl chains being unsaturated and a small headgroup would sort to negative curvature (Kamal et al., 2009). In line with this, several studies found an enrichment of phosphatic acid and other inverse conical shaped lipids at the neck of highly negatively curved membranes (Crowley et al., 2024; Putta et al., 2016; Zhukovsky et al., 2019; see also above references). Supplementing these *in vitro* studies, molecular dynamics simulations also show that lipids have the propensity to sort to a membrane region based on their intrinsic shape, sensing the spontaneous curvature of the membrane (Baoukina et al., 2018; Beltrán-Heredia et al., 2019; Koenig et al., 2023). Despite the wealth of studies in this area, the consensus is that lipid shape alone, while important, does not completely account for the measurable amount of lipid sorting required in live cells and that lipid-lipid or lipid-protein based interactions are necessary to amplify curvature-based sorting (Callan-Jones et al., 2011; Cooke and Deserno, 2006). This is also supported by our own work in live cells, where we find that, rather than the size of the SL headgroup, it is the structure of the ceramide domain with the presence or absence of the C14* motif that determines intracellular trafficking (Duclos et al., 2020; Garcia-Castillo et al., 2018; Schmieder et al., 2022; Chinnapen et al., 2012).

### 4.2 Nanodomain based lipid sorting

An alternative model or complementary concept to the above-presented idea of curvature-induced lipid sorting, is that curvature preference, or indeed, induction, could arise not due to the physical properties of singular lipid species but as an emergent behavior of lipid organization in the membrane. Under this umbrella, segregated membrane nanodomains would provide an explanation for the differential lipid distributions and consequently lipid territories observed throughout the cell. Rather than an individual lipid molecule sensing membrane curvature, membrane nanodomains, with their unique physical properties, e.g., their low bending modulus, would detect and/or induce curvature preferences (C. Black et al., 2014; Huttner and Zimmerberg, 2001). Small and local inhomogeneities in membrane composition within an organelle could thus give rise to vesicles with different lipid compositions. In addition, lipid inhomogeneity might minimize the energy costs of bending the membrane (Mukherjee et al., 1999; Mukherjee and Maxfield, 2000; van Meer and Sprong, 2008; Maxfield and McGraw, 2004; van Meer and Sprong, 2008). This interplay between curvature and phase separation has been demonstrated *in vitro* for membranes close to phase separation or demixing (Baumgart et al., 2003; 2005; Jülicher and Lipowsky, 1996; Lipowsky, 1993; Ogunyankin et al., 2013; Ogunyankin and Longo, 2013; Parthasarathy et al., 2006; Sorre et al., 2009; Veatch and Keller, 2003; Woodward et al., 2023). Roux et al. (2005), Ikonen (2008) for instance, were able to demonstrate that lipid tubes pulled from Lo-Ld phase separated vesicles were almost exclusively in the Ld phase, implicating that tightly packed SL, or PtdCho with fully saturated acyl chains, disfavor curvature. These results are consistent with those by Mukherjee et al. (1999) and our own work. We observed GSLs species with the C14* motif within endosomal recycling tubules - in the absence of cholesterol, implicating that rather than lipid shape, incorporation of GSLs with C14* motif into membrane nanodomains is the driver for the observed differences in GSL sorting (Schmieder et al., 2022).

Interestingly, work by the Lippincott-Schwartz group showed in an elegant study phase separated domains within the endosomal network by hypotonic swelling and cooling, implicating that small diffraction limited nanodomains could also exist within the endosomal network, not only the PM (King et al., 2020). This is in agreement with, as mentioned above, our results where we also observed segregated domains within endosomal vesicles (Schmieder et al., 2022).

### 4.3 Alternatives: proteins associated with lipid sorting

An important caveat in many experiments investigating lipid sorting is, that they are often conducted in cell-free, and thus, protein-free systems. However, it is unlikely that curvature or nanodomain-based lipid sorting are the sole driving forces for lipid sorting within a cell and that this process would occur without protein assistance. There are few examples of such protein-assisted lipid sorting. Convincing evidence comes from studies on caveolin and the transport of cholesterol to the PM (E. J. Smart et al., 1996). Such selective transport of cholesterol to the PM by caveolin would most likely affect the concomitant transport of cSL as well. This is in agreement with work by the Nichols group, which suggests that caveolin, apart from transporting cholesterol and SM to the PM, is also required for endocytic trafficking of excess SL to the lysosome (Shvets et al., 2015).

Apart from caveolin, this idea is supported specifically for cSL-binding toxins such as Shiga toxin or Cholera toxin or cSL-binding galectins (Chinnapen et al., 2012; Römer and Elling, 2011; Safouane et al., 2010, p. 2010; Sorre et al., 2009; Tian and Baumgart, 2009). Here, the geometry of multivalent binding of nanodomain cSL by the proteins induces lipid compression and membrane bending (Arumugam et al., 2021; Ewers et al., 2010; Kabbani et al., 2020; Koyama-Honda et al., 2020; Roemer et al., 2007; Watkins et al., 2019). This process facilitates uptake of the membrane-protein complexes through recruitment of cellular trafficking machinery and forms the basis of the glycolipid-lectin driven endocytosis (Simunovic et al., 2017; Day and Kenworthy, 2015; Rydell et al., 2013; Johannes et al., 2015; Simunovic et al., 2017). Intriguingly, cSL lipid structures required for these processes to occur, differ between the different clustering proteins. Simian virus 40 requires cSL with C14* motif to induce membrane invaginations and endocytosis (Ewers et al., 2010), Shiga toxin and Cholera toxin however require cSL that do not contain C14* motif (Roemer et al., 2007; Chinnapen et al., 2012).

**FIGURE 5:**
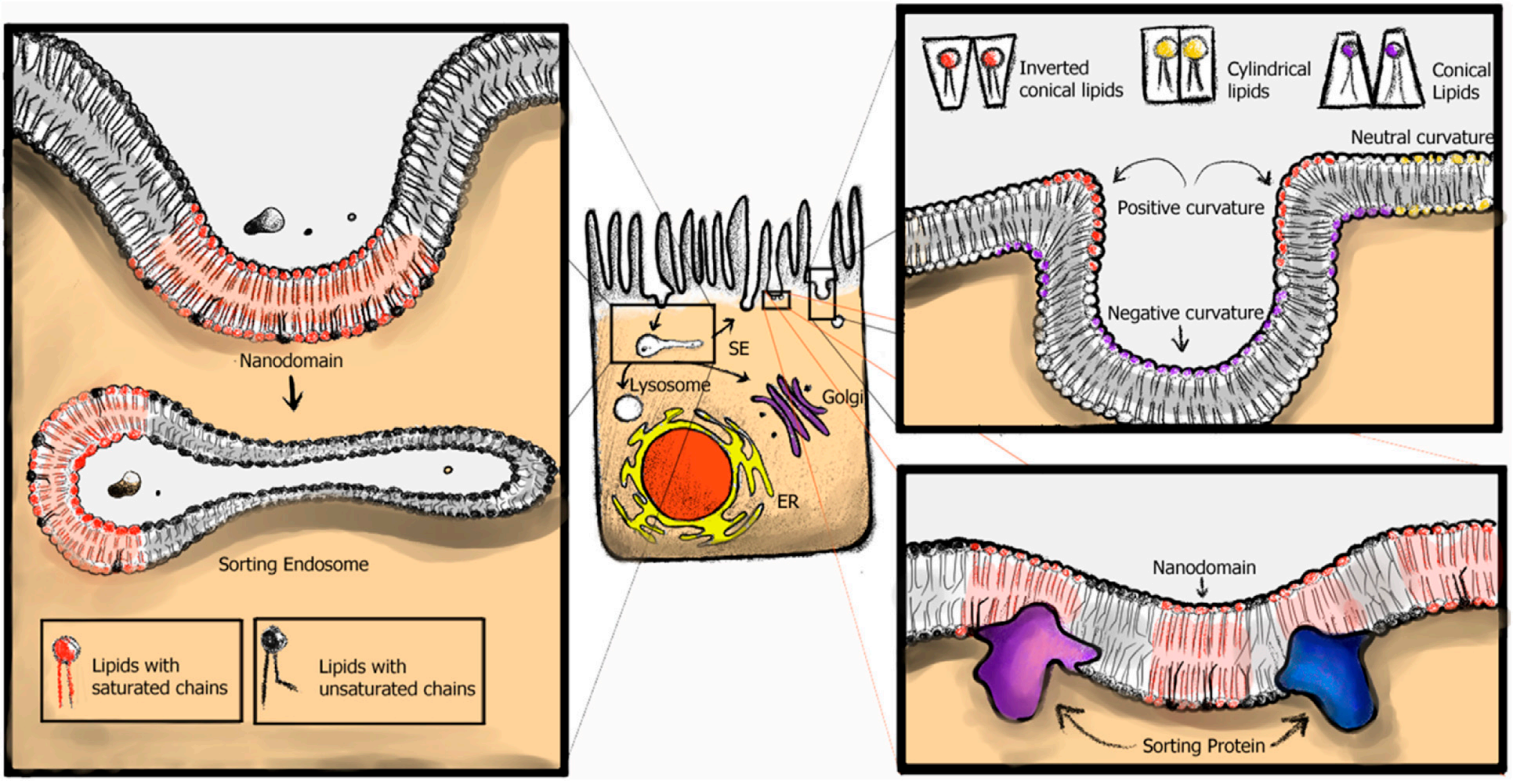
cSL sorting models. In cellular membranes curvature, nanodomain formation and cellular proteins recognizing local cSL heterogeneities contribute to their differential, subcellular sorting.

A concept for all the cSL sorting events mentioned in this review could be envisioned, where different proteins might immobilize and stabilize particular cSL distributions in the membrane and thus sort them in the process (Figure 5).

## 5 Open questions

We hypothesize that, most likely, SL sorting within the endomembrane system is a synergy of all three sorting mechanisms presented here. SL species that are structurally able to incorporate into membrane nanodomains are sorted as such, most likely aided by the cellular protein machinery. Conversely, SL that are unable to assemble into membrane nanodomains might experience sole curvature-based sorting mechanisms more strongly.

Most interesting is the recent discovery of bulk lipid exchange at membrane contact sites, which virtually interconnect all organelles. We envision that such bulk lipid exchange or the specific depletion/supplementation with certain lipid species could rapidly change membrane composition and fluidities in small organelles and thus drive demixing or curvature generation. An example is VPS 13C, mediating lipid exchange between the ER with the endosome, potentially supplying phospholipids to the endosome to ensure tubule formation (Suzuki et al., 2024).

Given the recent advances in the development of better lipid probes and membrane sensors, in combination with advancements in molecular dynamics simulations of more realistic membrane compositions and over longer time scales, it will be intriguing to see which endocytic and secretory proteins might function in the specific delivery of SLs to certain compartments.
